# Hybrid Epoxy-Alkyl Sol–Gel Coatings Reinforced with SiO_2_ Nanoparticles for Corrosion Protection of Anodized AZ31B Mg Alloy

**DOI:** 10.3390/gels8040242

**Published:** 2022-04-14

**Authors:** Emilia Merino, Alicia Durán, Silvia Ceré, Yolanda Castro

**Affiliations:** 1 Instituto de Cerámica y Vidrio (CSIC), Campus de Cantoblanco, 28049 Madrid, Spain; aduran@icv.csic.es; 2 Institute of Materials Science and Technology (INTEMA), University of Mar del Plata and National Research Council (CONICET), Av. Colón 10850, Mar del Plata 7600, Argentina; smcere@fi.mdp.edu.ar

**Keywords:** magnesium alloy, anodizing process, sol–gel, corrosion performance, hybrid inorganic-organic sol–gel coating

## Abstract

AZ31B Mg alloys were anodized at different potentials using an alkaline electrolyte. Then, an epoxy-alkyl silane sol reinforced with SiO_2_ nanoparticles was prepared by sol–gel and deposited on top of the optimized anodic layers. 1-Methyl imidazole was added to the sol to promote a partial epoxy ring aperture and improve the condensation degree of the inorganic network. The results showed the curing temperature affects the inorganic polycondensation of the organic-inorganic network; this effect was analyzed by ^29^Si and ^13^C solid-state NMR spectroscopy. Electrochemical impedance spectroscopy in 3.5 wt% NaCl solution revealed that the corrosion resistance is enhanced by the anodized process obtained for Mg alloy anodized at 100 V/2 min. However, a quick deterioration of the oxide film with immersion time was evident, showing a reduction of the protection efficiency (ηE%) of 76.5% after 16 h/immersion. The deposition of an epoxy-alkyl coating improved the ηE% up to 98.6% after 72 h/immersion. The proposed hybrid coating used for post-sealing the porous anodized Mg alloy looks like a good alternative protective barrier to control the corrosion process of Mg alloys. A suitable compromise between cross-linking network and curing temperature is necessary to obtain a good barrier coating.

## 1. Introduction

Magnesium and its alloys have become a hot research topic due to their excellent mechanical and physical properties such as low density, easy recyclability, and lightweight properties making them attractive for different industries (aerospace sectors, automotive and biomedical) [1]; especially for those industries where using lightweight metals on their products is indispensable to decrease their overall energy consumption [2]. Nevertheless, the wider applicability of Mg alloys has been limited by their high corrosion susceptibility [3].

Currently, various surface modification techniques are under study to improve the corrosion resistance property of light alloys [4,5]. Among these surface techniques, the electrochemical anodization process and sol–gel technique are considered and accepted as the most popular industrial processes, since they can provide relatively thick, hard, adherent, and corrosion abrasion-resistant oxide films [6]. A variety of commercial anodized coatings (Anomag, Keronite, Tagnite, HAE, Magoxid, Dow 17) have been considered to protect magnesium alloys against corrosion [7,8,9]. Dow 17 and HAE are reported as the most successful ones, but both employ electrolytes composed of toxic chromate and/or harmful fluorides [10].

To reduce the environmental impact and the health hazards concerned in handling fluoride/chromate-based baths, researchers have focused on seeking environmentally friendly solutions. Mizutani et al. [11] studied the anodization of pure Mg, AZ91, and AZ31 at various voltages (3, 10, and 80 V) in 1 mol L^−1^ NaOH and tested their corrosion resistance in the 0.1 wt% NaCl solution. They concluded that the films anodized at the lowest potential, mainly composed of magnesium hydroxide, showed the most effective anti-corrosion properties. This finding differs from Salman et al. [12], who reported that the film anodized at high voltage (100 V), mainly composed of Mg oxide, exhibited the most effective corrosion resistance properties. Since the results appearing in the literature show such contradictions, it was necessary to better understand the different phenomena acting in the anodizing processes at different working voltages as well as the corrosion mechanisms acting in the anodized coatings to establish the suitable conditions.

Although this approach is convenient to improve the corrosion resistance, current works show that a coating alone does not completely prevent the corrosion of Mg alloys [13,14]. In this sense, the combination of different deposition techniques could be the most effective method to mitigate corrosion damage. In our work, the use of the sol–gel technique as a post-treatment process was considered and studied to obtain a combined coating system. The most remarkable advantage of this technique is the opportunity to obtain organic–inorganic hybrid sol–gel coatings with desirable cross-linking structures and corrosion protection properties [15]. The properties of hybrid films are associated with their composition and density, which can be modified through the inclusion of a variety of organo-functional groups in the silane network [16]. Some authors have proposed the use of silanes precursors such as tetraethyl orthosilicate (TEOS), 3-glycidoxypropyltrimethoxysilane (GPTMS), and methyltriethoxysilane (MTES) to achieve good passive corrosion protection, avoiding the penetration of corrosive agents. For example, Guo et al. [17] used a TEOS, GPTMS, and triethylenetetramine (TETA) based sol–gel coating to improve the protection properties of the anodized layer on Mg AZ31B. Although the electrochemical response showed a rise in the corrosion resistance, the tests were performed in a non-aggressive environment (0.005 M of NaCl). On the other hand, Malayoglu et al. [18] observed an improvement in the corrosion performance for anodized AM60 and AM50 B Mg alloys post-sealing with a silica sol–gel film by using TEOS and MTES. In this case, MTES was added to provide a hydrophobic effect, associated with the -CH_3_ functionality group.

Our recent work has proposed an alternative pathway to achieve an interconnected structure film using GPTMS, colloidal SiO_2_ nanoparticles, TEOS, and 1-methyl imidazole [19]. The 1-methyl imidazole acts on the epoxy group, opening the ring and generating a highly crosslinking hybrid network, that can block the intrusion of the corrosive electrolyte and enhance the corrosion resistance performance of AZ31B Mg alloy. This dense 3D-cross-linked film with SiO_2_ nanoparticles free of a methyl group (as a network modifier) reduced the permeability of the film against the corrosive electrolyte and therefore increased the corrosion resistance of the material. However, the hybrid sol–gel film was not able to protect the anodized Mg alloy and rapid degradation of the system occurred after 8 h of immersion in NaCl.

So, the corrosion performance of silica sol–gel coating depends on the synthesis parameter such as the type of precursor and the curing process, including temperature and time. A suitable compromise between all of the parameters is necessary to obtain a good barrier.

In this paper, an epoxy-alkyl silane coating reinforced with SiO_2_ nanoparticles and catalyzed with 1-methyl imidazole (MI) was synthesized and deposited on an anodized Mg alloy. The effect of MI in the hydrolysis and condensation of metal alkoxides, which results in the formation of a hybrid network, was analyzed. The electrochemical impedance spectroscopy (EIS) technique was used to provide not only information about the instantaneous corrosion rate but also about the kinetic of the films in highly concentrated aqueous solution (3.5 wt% NaCl); in an attempt to mimic the most abundant and common corrosive agent on the earth (seawater).

## 2. Results and Discussion

After the pre-cleaned process, the Mg alloys were anodized by varying the potential from 3 to 100 V using a 1 mol L^−1^ NaOH aqueous electrolyte. Three different behaviors were observed depending on the anodizing potential. For 3 V, the dissolution of magnesium (reaction 1) and the alkalization of the media (reaction 2) take place and the film starts to grow since the Mg^2+^ ions react with OH^-^ ions to yield Mg (OH)_2_. (reaction 3).

(1) $$\mathrm{Mg}\to {\mathrm{Mg}}^{2+}+2e^{-}\;$$

(2) $$2H_{2}O+2e^{-}\;\to H_{2}+2{\mathrm{OH}}^{-}\;$$

(3) $${\mathrm{Mg}}^{2+}+2{\mathrm{OH}}^{-}\to \mathrm{Mg}{\left(\mathrm{OH}\right)}_{2}$$

At 10 V, a new direct electrochemical oxidation of Mg to MgO (reaction 4) also takes place.

(4) $$\mathrm{Mg}+2{\mathrm{OH}}^{-}\to \mathrm{MgO}+H_{2}O+2e^{-}$$

For working voltages of 30 V and 70 V, a slight evolution of hydrogen was observed during the anodization process. However, when the working voltage changes to 100 V, a new phenomenon (called uniform sparking) appears. The sparking arc occurs by the action of a strong electric field (break down event) which vanishes with time. After 2 min of anodizing time, a deterioration of the coating is clear, indicating that anodizing times longer than 2 min are not adequate for an anodizing voltage of 100 V.

Yahalom et al. [20] reported that the temperature of the sparking arc during the anodization procedure could reach temperatures above 1000 °C, provoking the increase in temperature of the alloy surface. This high temperature could induce a dehydration process (reaction 5) as reported by Feitknecht and Braun [21], increasing the amount of MgO in the surface film.

(5) $$\mathrm{Mg}{\left(\mathrm{OH}\right)}_{2}\to \mathrm{MgO}+H_{2}O\;\;$$

**Figure 1:**
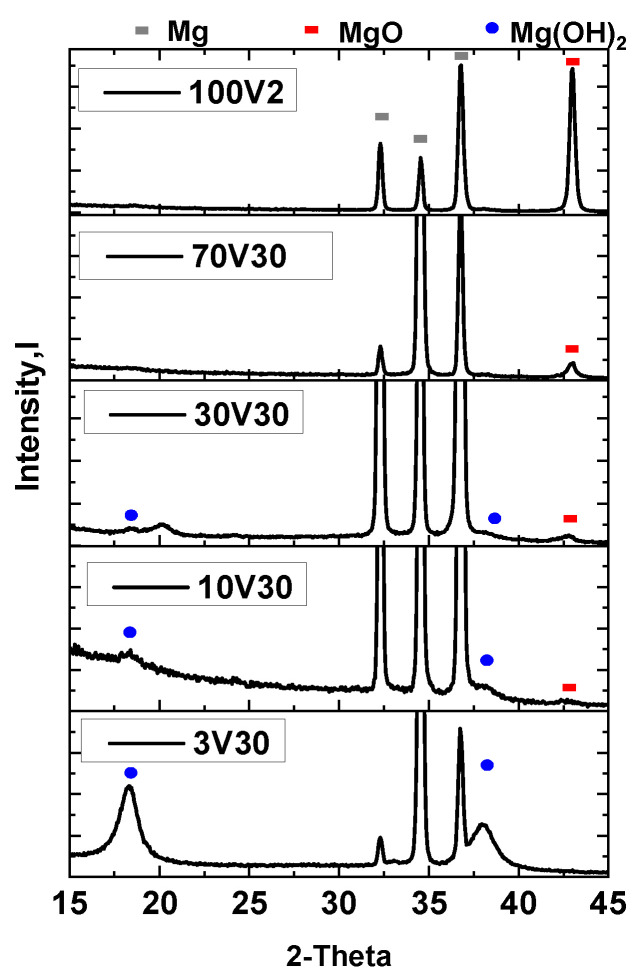
XRD patterns of anodized AZ31B alloy coatings obtained at different working voltages.

X-ray diffraction patterns of anodized magnesium alloy obtained at different voltages are depicted in Figure 1.

X-ray diffraction patterns show that as the anodizing potential increases, the intensity of Mg(OH)_2_ diffractions peaks (●) decreases, and MgO peaks (■) increase. Therefore, anodic films obtained at low voltage (3 V) are enriched in Mg(OH)_2_, while coatings obtained at 100 V contain more MgO. According to Lei et al. [22], a high proportion of MgO in the oxide coating could protect more of the Mg substrate from corrosion attack than Mg(OH)_2_. These results are congruent with previously proposed mechanisms and with the literature reports [12]. In conclusion, the applied voltage governs the relative concentrations of magnesium oxide and hydroxide in the anodic film.

**Figure 2:**
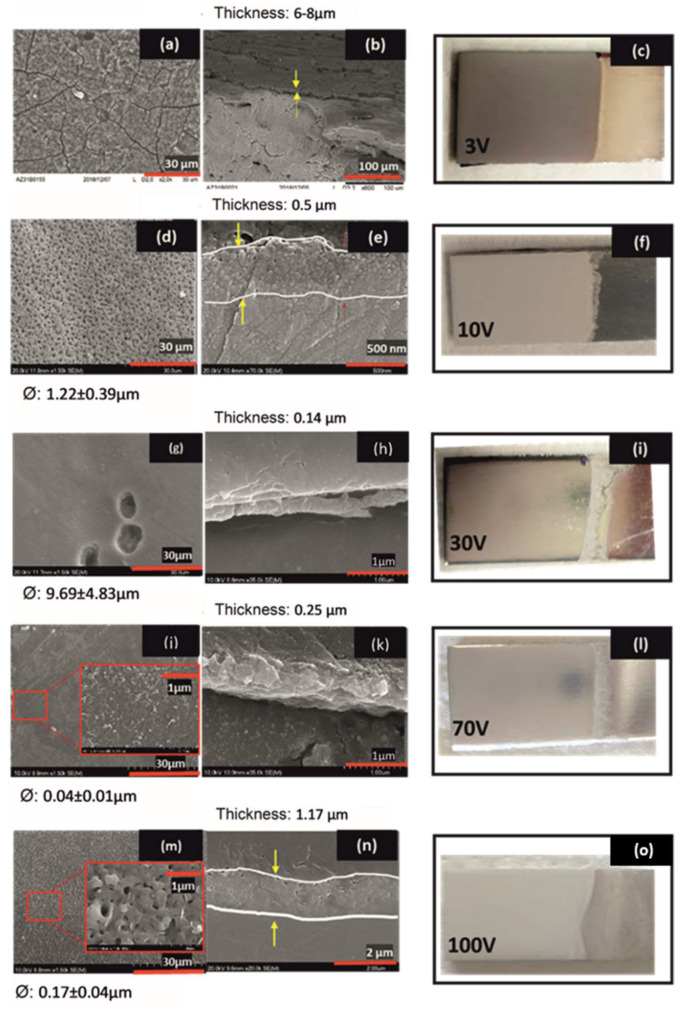
FESEM images of the morphology, cross-section, and aspect of the anodized AZ31B alloys samples (**a**–**c**) 3V30, (**d**–**f**) 10V30min, (**g**–**i**) 30V30, (**j**–**l**) 70V30, and (**m**–**o**) 100V2 in 1 mol L^−1^ NaOH electrolyte; ⌀: Average of the diameter pores size.

Figure 2 shows the surface and cross-section morphology of anodized films obtained at different potentials. For the anodizing process at 3 V (3V30), a homogeneous and cracked film appears (Figure 2a). The cross-section shows an anodized layer with a coating thickness of around 7 µm (Figure 2b). This morphology is associated with a dark greyish coating (Figure 2c).

A homogeneous and crack-free anodized porous film is obtained by increasing the anodization potential up to 10 V (10V30). FESEM images reveal a uniform porous coating structure with a pore size of 1.2 ± 0.4 µm in diameter (Figure 2d) and a coating thickness of ~0.5 µm (Figure 2e). The surface morphology corresponds to the light greyish aspect shown in Figure 2f.

At higher anodizing potentials, 30 V (30V30) and 70 V (70V30), crack-free surfaces with non-uniform morphology are obtained as shown in FESEM images (Figure 2g,j). However, for the anodizing process at 100 V for 2 min (100V2), a homogenous flower-shaped appearance is observed (Figure 2m), corresponding to the vigorous sparking conditions.

At 30 V, 70 V, and 100 V, the average pore size was around 9.7 ± 4.8 µm, 40 ± 10 nm, and 170 ± 40 nm, and the thickness of the anodized film was 0.14 µm, 0.25 µm, and 1.17 μm (Figure 2h,k,n)), respectively. The surface films anodized at 30 V and 70 V exhibited a bright reflective finishing (Figure 2i,l), while 100 V sample presents a dense white color (Figure 2o).

The contact angle for anodized AZ31B substrates was measured. The highest contact angle was obtained for the as-received substrate (93.5 ± 4.5°) and the lowest contact angle for the 3V30 (immediately adsorption) and 100V2 (35.7 ± 1.2°) samples. The low wettability of as-received substrates is associated with the higher roughness surface of 2.2 µm, susceptible to trapped air in the micro-nanostructure, increasing the water contact angle [3]. On the other hand, the higher wettability of the 3V30 and 100V2min samples is associated with the cracked Mg(OH)_2_ enriched coating and with the porous-smoother surface, respectively.

**Figure 3:**
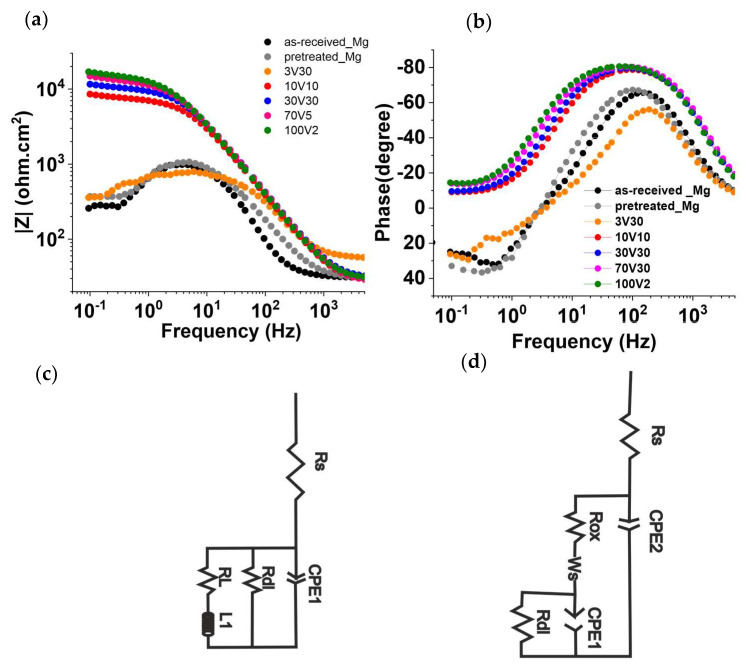
(**a**,**b**) Bode plot for anodized coatings at different working voltages. Equivalent circuit for (**c**) as-received, pretreated, and anodized film at 3V30 and (**d**) anodized films at 10V10, 30V30, 70V30, and 100V2 after 2 h of immersion in 3.5% wt NaCl solution.

Figure 3a,b shows the Bode plots of the anodized films obtained at 3V30, 10V30, 30V30, 70V30, and 100V2, along with the as-received and pre-cleaned substrates. EIS data were fitted using the equivalent circuits proposed in Figure 3c,d.

In general, the equivalent circuits include the electrolyte resistance (Rs), the charge transfer resistance (Rdl), the electrical double-layer capacitance (CPE1), the electrolyte resistance into the coating pores (Rox), the constant phase element of the anodized film (CPE2), a finite length Warburg short-circuit (Ws), and the resistance (RL) of an inductance (L1) process. The CPE behavior is generally attributed to a distribution of time constant and is calculated using the following formula (Equation (1)):

(6) $$\mathrm{CPE}=1/Q{\left(j\omega \right)}^{\alpha }$$

where Q is a parameter independent of frequency having units of Ω^−1^cm^−2^s^α^ for α≠1, and F cm^−2^ when α = 1, and α may vary from −1 to 1, with 1 as the ideal capacitor [23].

The Bode plot shows that the impedance spectrum of anodized film at 3V30min has a similar trend as the as-received and pretreated AZ31B specimens. These spectra show a decreasing trend in the impedance magnitude lZl and phase angles values closed to 40° at the low frequencies domain (f < 1). This behavior relates to the presence of an inductive loop (RL, L1) at low frequencies, included in the representation of the equivalent circuit in Figure 3c. The inductive behavior can be associated with the breakdown of the protective coating [24] or with the relaxation processes of adsorbed species such as Mg(OH)_ads_^+^ or Mg(OH)_2_ onto the electrode surface as a consequence of the pitting corrosion process [25,26]. The presence of cracks in the anodized coating at 3V30 facilitates the fast diffusion of (Cl^−^) ions through the coating, easily reaching the inner Mg matrix. Further, the main composition of the anodized coating, Mg(OH)_2_, provides relatively low corrosion resistance to the AZ31B alloy since the chloride ions can transform the Mg(OH)_2_ to MgCl_2_, a more soluble species [2], promoting the dissolution of the Mg alloy [22]. (reaction 6)

(7) $$\mathrm{Mg}{\left(\mathrm{OH}\right)}_{2}+2{\mathrm{Cl}}^{-}\to {\;\mathrm{MgCl}}_{2}+2{\mathrm{OH}}^{-}$$

In this case, neither the etching process nor the Mg(OH)_2_ coating obtained after the anodizing process is enough to obtain a good corrosion barrier.

For 10V30min, 30V30min, 70V30min, and 100V2min samples, a different trend is observed in the low frequencies domain (f < 1), with a significant increase in the impedance modulus lZl. In this case, the equivalent circuit (Figure 3d) included a finite length Warburg short-circuit (Ws) and two-time constants (Rox, CPE2, Rdl, and CPE1).

The time constant at high frequency is related to the porous anodized layer (Rox, CPE2), and the time constant at low frequency is attributed to the internal layer structure of the anodized coating, which corresponds with a charge transfer metal–film interface resistance (Rdl), and a capacitive element CPE1. The Warburg element in the equivalent circuit corresponds to the diffusive process occurring through the anodized films in the absence of corrosion products. The change from adsorption to mass transport processes likely suggests that the anodized coating blocks the easy diffusion of the electrolyte to the substrate, due to the crack-free coating morphology [27]. Considering the proposed equivalent circuit Figure 3c,d, the total corrosion resistance for each system and Rp (polarization resistance) values were calculated as the sum of all the faradaic resistance (Table 1). Additionally, to compare the pseudo-CPE capacitance with the classic capacitance, the effective capacitance (C_eff_) was also calculated using the Hirschorn et al. [28] model based on the Brug’s equation (Equation (2)) and considering a parallel surface distribution [29].

(8) $$C_{\mathrm{eff}}={\mathrm{CPEcoat}}^{\begin{array}{c} \left(\frac{1}{\alpha \mathrm{coat}}\right) \end{array}}{\left(\frac{\mathrm{Rs}*\mathrm{Rp}}{\mathrm{Rs}+\mathrm{Rp}}\right)}^{\frac{1-\alpha \mathrm{coat}}{\alpha \alpha \mathrm{coat}}}$$

Both the Rp and C_eff_ values for each condition are also included in Table 1. The polarization resistance for the as-received, pre-cleaned, and the anodized film obtained at 3V30 min showed the lowest values, around 200 Ω cm^2^. However, an increment of Rp values appears when increasing anodizing potential, following the order: 100V2 > 70V30 > 30V30 > 10V30. Coated specimens with high polarization resistance values present better corrosion resistance [30]. Therefore, the best corrosion resistance performance was obtained for 100V2 coating.

Although the 100V2 sample showed the highest Rp values (13,557 Ω·cm^2^), non-significant differences can be observed along with the C_eff_ values since all coated samples showed values in the order of 10^6^ F cm^−2^. This means that in general the oxide coatings obtained at different anodizing potentials and times show similar electrolyte uptake phenomena (electrolyte penetration/adsorption) and therefore, the corrosion process slows down by a double effect of a decrease in charge transfer area associated with the small pore size obtained at a higher anodizing voltage (between 40 nm–0.17 µm for 70 and 100 V, respectively), and/or the low anodic dissolution of the coating due to the presence of MgO in the coating composition, more stable than Mg(OH)_2_, regardless of the differences in oxide thickness. These results differ from Mizutani et al. [11] but are similar to those of Salman et al. [12], where the presence of MgO is decisive to control the corrosion processes much better than oxide film composed of Mg(OH)_2_.

Although the anodized coating obtained at 100V2 increases the corrosion resistance of the naked substrate, its corrosion protective performance is still not comparable to other anodized systems obtained using more complex electrolytes. For example, Ying Long et al. [31] reported a corrosion resistance of 32,205 Ω·cm^2^ for an anodized coating obtained using an alkaline electrolyte that contains Na(OH) and/or Na_2_SiO_4_ and alumina nanoparticles as additives. Therefore, to improve the corrosion resistance of the AZ31B alloy, in this work, the anodized specimens were sealed using an epoxy-alkyl hybrid silica sol–gel (SGMI) prepared following the process described in the experimental section. A homogeneous and transparent SiO_2_ sol, with an adequate viscosity of around 3.6 mPa.s and a neutral pH was obtained.

**Figure 4:**
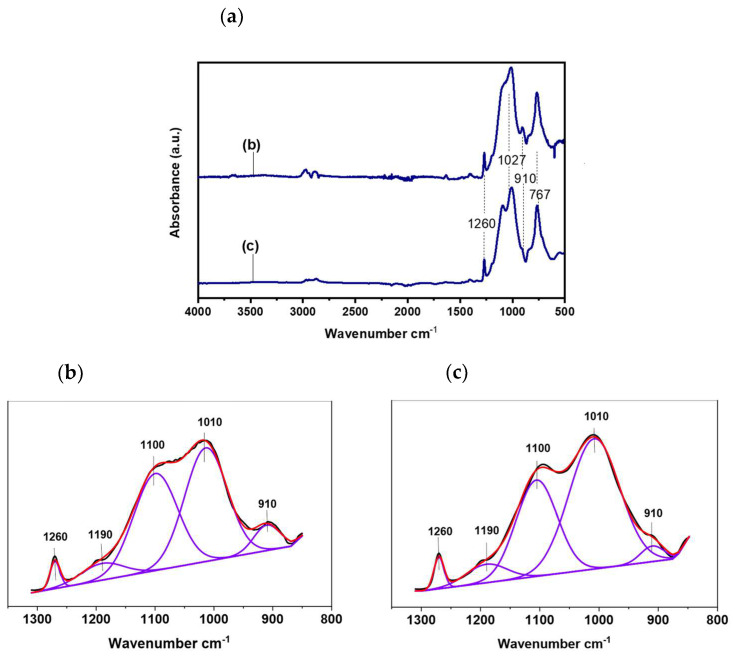
FTIR spectra of the silica sol–gel without MI and with MI (**a**) and the corresponding integral of the deconvoluted peaks in the region 1350–1850 cm^−1^ (**b**,**c**).

FTIR measurements were performed to study the effect of adding MI in the organic-inorganic polymerization of the hybrid sol. Figure 4a) shows the FTIR spectra of hybrid silica sols prepared with and without the addition of MI, and Figure 4b,c show the corresponding FTIR spectrum and its deconvoluted peaks in the region between 1350 and 850 cm^−1^. For both spectra, the characteristic peak of the Si–O–Si bond at 1027 cm^−1^ was identified, confirming that the inorganic polymerization reaction took place [32]. A band at 1260 cm^−1^ is ascribed to Si–C of the CH_3_ group of MTES precursor, and a peak at 910 cm^−1^ is associated with the stretching vibration of the epoxy ring [32]. The broad peak around 1250–850 cm^−1^ was deconvoluted; the band at 1270 cm^−1^ assigned to Si-CH_3_ was chosen to normalize the integrated area (Table 2). It is possible to identify two bands at 1100 cm^−1^ and 1010 cm^−1^ associated with the longitudinal optic (LO) and the transversal optic (TO) modes of Si-O-Si vibrations, respectively. The ratio of the LO and TO modes is associated with the porosity of the silica network [33,34]. As observed, the ratio LO/TO decreases from 0.88 to 0.60 with the addition of MI indicating the increment of inorganic condensation and a less porous structure. Furthermore, the ratio between the epoxy (910 cm^−1^) and Si-CH_3_ bands also decreases (approximately 50%) with the addition of MI, indicating a partial opening of the epoxy ring groups to form a more cross-linked network.

**Figure 5:**
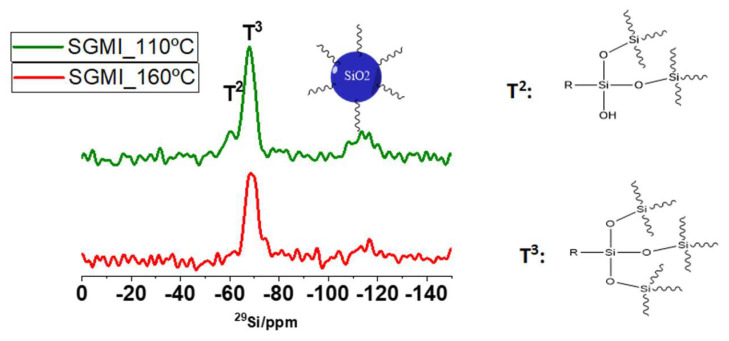
^29^Si NMR spectra taken from cured SGMI sol at 110 °C and 160 °C in the region of 0 and −150 ppm.

In order to evaluate the effect of the sintering temperature on the structural features of the hybrid network, the silica sol was cured at 110 °C and 160 °C and characterized by solid-state ^29^Si and ^13^C NMR. Two intense peaks at −70 and −60 ppm are observed (Figure 5) and assigned to T^3^ (RSi(OSi)_3_), and T^2^ (RSi(OSi)_2_OH) units (R: –CH_3_, propyl chain of GPTMS). In the range between −100 and −130 ppm a broad band, which corresponds to Q^n^ (Si(OSi)_4_) of the Ludox nanoparticles, was also observed. The absence of T^0^ and T^1^ units indicates a high polycondensation of the silica network. It is noteworthy to observe that the relative intensity of the T^2^ peak decreases from 110 °C to 160 °C, meaning that the degree of condensation of the inorganic phase increases. Therefore, the formation of a cross-linked inorganic network is evident when the film is treated at 160 °C. On the other hand, polymerization of epoxy groups was also monitored by ^13^C solid-state CPMAS-NMR (not shown) and there is no evidence of an increase in the organic polymerization with temperature.

**Figure 6:**
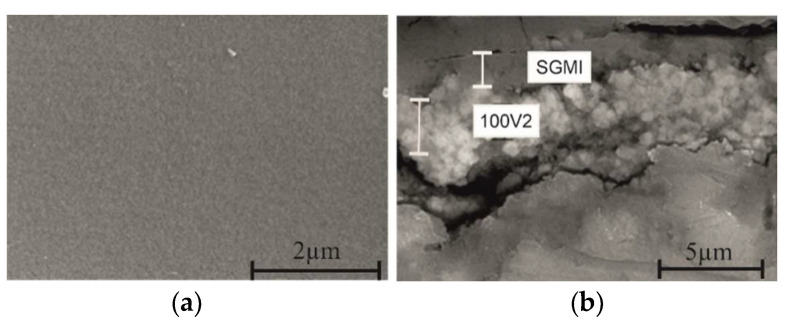
FESEM surface morphology (**a**) and cross-sectional (**b**) images of sealed system heat treatment at 110 °C/h.

Thus, Mg alloy anodized at 100V2 was sealed using the SiO_2_ sol and heat-treated at the two different temperatures, 110 °C and 160 °C for one hour. Figure 6 shows the FESEM images of surface morphology and cross-section of the sealed system sintered at 110 °C/1h. Homogeneous, smooth, and crack-free coated surfaces are obtained with a total thickness of ~3.2 µm (anodized and silica coatings) at 110 °C and 160 °C, respectively. The contact angle measured for the sealed system was 78.8 ± 4.2°, higher compared to 35.7 ± 1.2° for 100V2 oxide coating.

**Figure 7:**
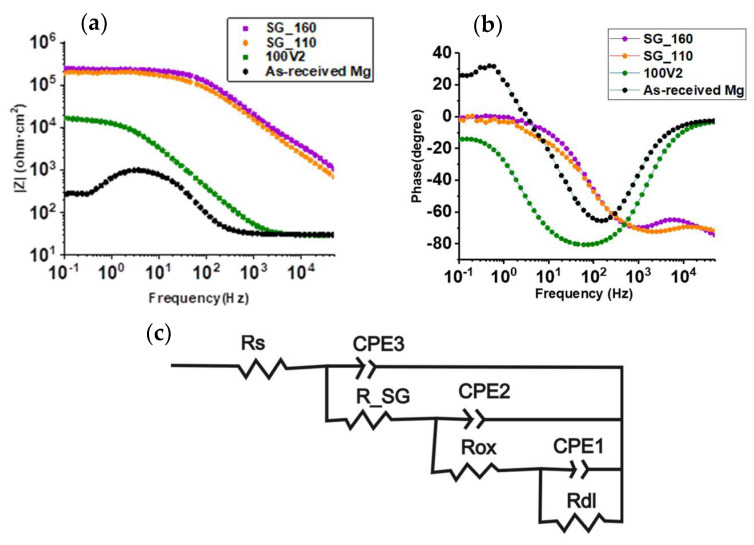
(**a**,**b**) Bode plots for the anodized coating (100V2min), SG_160, SG_110 and as-received Mg alloy after 2 h of immersion in 3.5% wt NaCl solution. (**c**) equivalent circuit for SG_110 and SG_160.

The corrosion resistance properties of SGMI_160 and SGMI_110 systems were evaluated by electrochemical impedance spectroscopy (EIS), Figure 7. Rp and effective capacitance (C_eff_) values were also calculated considering the equivalent circuits presented in Figure 7c, which include the electrolyte resistance (Rs) and tree-time constants (Table 1). R_SG and CPE3 represent the resistance and the capacitance related to the hybrid organic–inorganic coating. Rox and CPE2 are the resistance and the capacitance of the anodized film, and Rdl and CPEdl are attributed to the charge transfer process in the film/substrate interface region. Warburg element impedance disappears from the equivalent circuits unlike the proposed anodized circuit (Figure 3d), indicating that silica sol–gel coating seals the pathways for diffusion of corrosive species through the anodized coating. The coating protection efficiency (ηE) obtained by the EIS test was calculated (Table 1) by the following relationship (Equation (3)):

(9) $$\eta E\%=\frac{Rp\left(\mathrm{coat}\right)-Rp}{Rp\left(\mathrm{coat}\right)}\times 100$$

where Rp(coat) and Rp are the values of polarization resistance of the anodic film/hybrid silica sol–gel system and the bare Mg alloy, respectively.

Better corrosion resistance properties are obtained for both SGMI_160 and SGMI_110 systems compared with 100V2 anodized sample and bare Mg alloy since the polarization resistance (Rp) values are one order of magnitude higher than the 100V2 anodized sample and three orders of magnitude higher than bare Mg alloy. These results indicate that the proposed sealed system is more effective against corrosion than the use of a single-anodized layer system.

The highly cross-linking silica coating reduces the penetration of the electrolyte through the anodized coating (water uptake), which is in good agreement with the lower C_eff_ value of the sealed system, three orders of magnitude lower (10^−9^ F·cm^−2^) than the anodized Mg alloy (10^−6^ F·cm^−2^).

**Figure 8:**
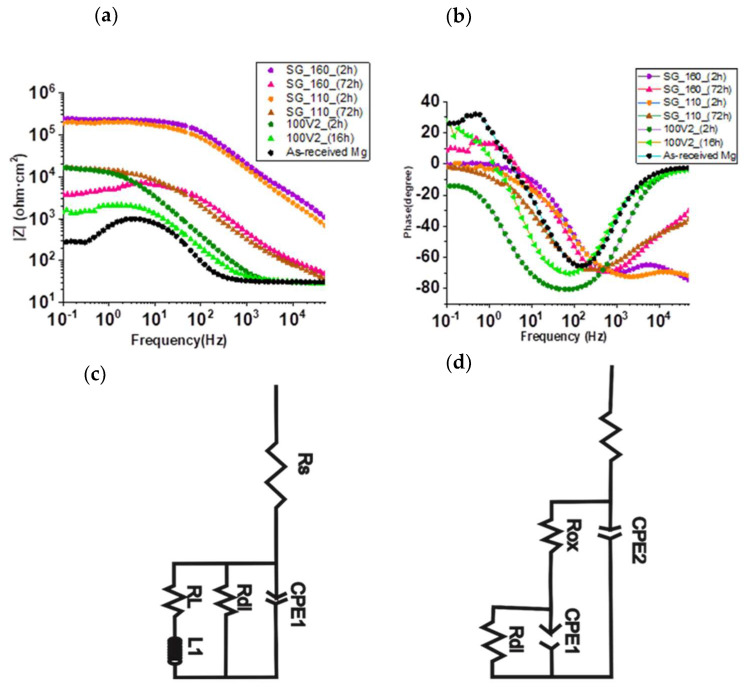
(**a**,**b**) Bode plots for the anodized coating (100V2min), SG_160, SG_110, and as-received Mg alloy at different immersion times in 3.5% wt. NaCl solution. (**c**) equivalent circuit for 100V2min and SG_160 after 16 h and 72 h immersion time, respectively, and (**d**) equivalent circuit for SG_110 after 72 h of immersion.

Further, the corrosion behavior of SGMI_110 and SGMI_160 samples were analyzed as a function of immersion time in 3.5% NaCl and compared to 100V2 oxide film. Bode and Phase Angle plots (Figure 8a,b) show that the impedance magnitude lZl value at the low-frequency domain (f < 1 Hz) decreases with the immersion time, indicating a diminution of the corrosion resistance properties of the coating systems. For the 100V2 sample, the Rp value drops significantly from 13,557 Ω cm^2^ to 884.2 Ω cm^2^ after 16 h of immersion showing a loss of the protection efficiency from 98.4% to 75.5%. Short-term corrosion protection is observed for the anodized coating associated with the fast electrolyte uptake through the hydrophilic porous oxide film. On the other hand, the polarization resistances for SGMI_160 and SGMI_110 systems were 1546 Ω cm^2^ and 14,903 Ω cm^2^ after 72 h; one or two orders of magnitude higher than in the case of the anodized coatings (884.2 Ω cm^2^) after 16 h of immersion. As can be seen, longer-term corrosion protection was obtained for the less cross-linking structural network (SGMI-110), with a protection efficiency of 98.6 % after 72 h of immersion compared to SGMI-160. The quicker decay of the corrosion resistance performance of SGMI-160 can be associated with the internal stresses and higher rigid film structure that led to the formation of micro-cracks (not observed by FESEM) at high heat-treatment temperature.

These results suggest that MgO enriched coating can be considered a good first approach to block the corrosion process of Mg alloys. However, higher corrosion activity and consequently low polarization resistance are observed after the beginning of the immersion tests, indicating that the anodized coating does not provide long-term corrosion protection. Sealing of the anodized coating with a silica sol–gel improves the corrosion resistance properties of Mg alloys. The control of the sol–gel synthesis and the right choice of the treatment temperature allow for improving the corrosion resistance and the stability of the protective system with time.

## 3. Conclusions

In this study, the anodizing process of AZ31B Mg alloy was studied as a function of potential and time, evaluating the effect of these conditions on the corrosion performance of the anodized samples. Relevant differences were identified related to the morphology and composition of the anodized films. The anodized film obtained at 3 V for 30 min exhibited a cracked morphology, mainly composed of Mg (OH)_2,_ showing the worst corrosion resistance performance. As the anodized potential increases, cracked-free and MgO enriched coating appear. The most MgO-enriched and dense anodized coating (small pore size) was obtained when the AZ31B Mg alloy was anodized at 100 V for 2 min; this coating showed a higher value of Rp and a non-significant difference in the C_eff_ values with respect to the other anodized coatings indicating that the improvement in the corrosion resistance can be likely attributed to the small pore size and the existence of MgO-enriched coatings. However, the coating barrier properties degrade fast with the immersion time. The sealing of the anodized sample using the epoxy-alkyl sol–gel coating reinforced with SiO_2_ nanoparticles decreases the effective capacitance (C_eff_) value and slows down the corrosion rate of the Mg alloys in concentrated NaCl solution. The MI addition promotes the aperture of the epoxy ring and a more cross-linked network with a less porous structure, thus increasing the anodized Mg alloy corrosion resistance. The use of different curing temperatures in terms of corrosion performance did not show a remarkably different electrochemical contribution during the first two hours of immersion.

The curing temperature plays an important role in the corrosion protection performance with immersion time. Although better corrosion performance is expected with higher condensed Si–O–Si structure film, internal stresses and the degree of network rigidity could lead to a faster decay of the corrosion resistance performance of the silane coating with time.

## 4. Materials and Methods

Commercially available AZ31B Mg alloy with composition: 10.1% Al, 0.3% Mn, 1.0% Zn, <0.5% Fe, and Mg balance (Dugopa S.A, Madrid, Spain) was cut to a size of 5 × 2 cm^2^. To remove oil and surface imperfections, the AZ31B Mg samples were dipped in 2 vol% H_3_PO_4_ (85%, Aldrich, Madrid, Spain) and 10 vol% HNO_3_ (65%, VWR, Barcelona, Spain) acid solution for 20 s, then rinsed with water and finally neutralized with 5 wt% NaOH (99%, Scharlab, Barcelona, Spain) solution.

### 4.1. Preparation of the Anodizing Coating

Anodization experiments were performed in an aqueous electrolyte of 1 mol L^−1^ NaOH (99.9%, Scharlab, Barcelona, Spain) at room temperature using a DC power supply (Magna Power, 12 A, 500 V, Flemington, NJ, USA). A platinum foil was fixed as the cathode and the pre-cleaned AZ31B substrate as the anode.

Different anodizing tests were performed by varying the potentials from 3 to 100 V. All the samples were anodized for 30 min except the ones at 100 V that were treated for 2 min. The samples were labeled as XVY, where XV corresponds to the anodizing potential (3, 10, 30, 70, and 100 V) and Y to the anodization time (2 and 30 min).

### 4.2. Preparation and Deposition of the Epoxy-Alkyl-Silane Coating

A modified silane sol was synthesized at room temperature by mixing 0.17 mol of 3-(glycidyloxypropyl) trimethoxysilane (GPTMS, ABCR, 98%, Karlsruhe, Germany), 0.5 mol of methyltriethoxysilane (MTES, Aldrich, 99%, Madrid, Spain), 0.33 mol of colloidal SiO_2_ nanoparticles suspension (Ludox-4S, Aldrich, aqueous suspension 40 wt%, particle size 20 nm, pH9, Madrid, Spain), and 0.014 mol of nitric acid HNO_3_ (VWR, 65%, Barcelona, Spain). After the reaction, 0.014 mol of 1-Methylimidazole (MI, Aldrich, 99%, Madrid, Spain) diluted in ethanol (EtOH, Panreac, 99.8%, Madrid, Spain) was incorporated to obtain a transparent sol.

The viscosity of the sol was controlled with an A&D Vibro Viscosimeter SV-10 equipment (Malvern Panalytical, Madrid, Spain), and pH was measured by a pH paper indicator (VWR, Barcelona, Spain).

The structural changes in the silica sol preparation were analyzed by Fourier Transformed Spectroscopy (FTIR) (Perkin Elmer Spectrum 100 spectrometer, PerkinElmer, INC, Madrid, Spain), a spectrometer with an attenuated total reflectance accessory. FTIR spectra were measured with a resolution of 4 cm^−1^ and 8 scans for each measurement.

The anodized samples were coated with the sol using a withdrawal rate of 30 cm/min. After the deposition, the samples were cured at two temperatures between 160 °C and 110 °C for 1 h. The samples were labeled as SGMI_160 and SGMI_110 for the system composed of the oxide coating plus the silica sol–gel coating heat-treated at 160 °C and 110 °C, respectively.

Solid-state ^29^Si MAS and ^13^C CPMAS-NMR spectra were recorded using a Bruker Avance-400 pulse spectrometer equipped with a fast Fourier transform unit. Samples were spun at 10 kHz around an axis inclined 54 ° 44′ with respect to the external magnetic field. The resonance frequencies used were 79.5 and 100.63 MHz (9.4 T magnetic field). ^29^Si MAS spectra were recorded after irradiation of samples with a π/2 (5-µs) pulse. In order to avoid saturation effects, the recycle delay time used was 10 s. A contact time of 2 ms and a recycle delay of 5 s were used in ^13^C CPMAS-NMR experiments. All measurements were taken at room temperature with TMS (tetramethylsilane) as an external standard. The error in chemical shift values was estimated to be lower than 0.5 ppm.

### 4.3. Characterization of the Coatings

The surfaces morphology of samples was characterized using Field Emission Scanning Electron Microscopy (FESEM, Hitachi S4700, Japan, Tokyo). The images obtained by FESEM were evaluated using ImageJ software (U. S. National Institutes of Health, Bethesda, Rockville, MD, USA). The surface roughness (Ra-parameter) was determined using a Zeta 20 optical profiling microscope (KLA Corporation, Milpitas, CA, USA) set at 20× magnification and a resolution of 0.1 nm. A ‘Drop Shape Analysis System’ Kruss DSA 100 system (Kruss, Hamburg, Germany) was used to measure the water contact angle. An analytical X’Pert PRO theta/theta diffractometer (Malvern Panalytical, Madrid, Spain) was used to analyze the crystallographic structure of anodized films. A grazing angle of 0.5° and 2θ range of 10–50° with a step size of 0.05° and accumulation time of 20 s per step and Cu-Kα radiation (λ = 0.15418 nm) as the excitation source was used to perform the experiment.

The electrochemical characterization was developed in a Gamry FAS2 electrochemical unit (Gamry, United Kingdom, Warminster) with DC and AC signals. Electrochemical Impedance Spectroscopy (EIS) was measured using a saturated calomel electrode (SCE, Radiometer, Hach Lange GmbH, Germany, Düsseldorf) as the reference electrode, the metal samples as the working electrode, and the platinum wire as the counter electrode. The testing was carried out in 3.5 wt% NaCl electrolyte solution considering an area of 0.78 cm^2^. EIS results were conducted in a frequency range of 10^5^ Hz to 0.1 Hz with an application of an AC voltage at the open circuit potential with a sinusoidal amplitude of 10 mV rms. After each sample was measured three times, the most representative measurement was plotted. The impedance plots were fitted in the Zview 2.0 software using a compatible equivalent circuit that simulates the corrosion behavior of the anodized and non-anodized samples.

## Figures and Tables

**Table 1:** Impedance parameters of anodized coated specimens obtained via the EIS data fitted with the equivalent circuit.

Sample	R_SG	Rox	RdL	RL	CPE1		CPE2		CPE3		Rp	C_eff_	ηE
	Ωcm^2^	Ωcm^2^	Ωcm^2^	Ωcm^2^	CPE1	α1	CPE2	α2	CPE3	α3	Ωcm^2^	Fcm^−2^	%
100V2	-	11,873 (±65)	1284 (±62.19)	-	2.5 × 10^−5^ (±2. × 10^−6^)	1	5.2×10^−6^ (±1.0×10^−8^)	0.95 (±2.4×10^−4^)	-	-	13,557	3.32 × 10^−6^	98.5
100V2_(16h)	-	-	2190 (±47.77)	1483 (±107.36)	1.3 × 10^−5^ (±6.46 × 10^−7^)	0.91 (±7.25 × 10^−3^)	-	-	-	-	884.2	6.21 × 10^−6^	76.6
70V30	-	10,127 (±299)	1340 (±275)	-	2.2 × 10^−5^ (±9.9 × 10^−6^)	1	4.9 × 10^−6^ (±5.5 × 10^−8^)	0.95 (±0.01)	-	-	11,467	3.11 × 10^−6^	98,2
30V30	-	8025 (±89)	777.4 (±82.27)	-	2.2 × 10^−5^ (±5.9×10^−6^)	1	4.8 × 10^−6^ (±1.8 × 10^−8^)	0.96 (±5.0 × 10^−4^)	-	-	8802	3.34 × 10^−6^	97.6
10V10	-	6019 (±56)	561.3 (±45.25)	-	2.4 × 10^−5^ (±4.8 × 10^−6^)	1	5.3 × 10^−6^ (±1.7 × 10^−8^)	0.96 (±4.23 × 10^−4^)	-	-	6580	3.69 × 10^−6^	96.8
3V30	-	-	684.2 (±7.4)	345.1 (±20.43)	7.4 × 10^−6^ (±3.8 × 10^−7^)	0.94 (±7.22 × 10^−3^)	-	-	-	-	229.3	4.43 × 10^−6^	9.6
SG_110	14,365 (±1202.5)	152,350 (±2074)	34618 (±2001)	-	4.4 × 10^−7^ (±5.3 × 10^−8^)	1	1.5 × 10^−9^ (±1.0 × 10^−10^)	1	3.7 × 10^−8^ (±1.0 × 10^−9^)	0.83 (±2.4 × 10^−3^)	201,333	2.21 × 10^−9^	99.9
SG_110_ (72h)	-	72.58 (±10.38)	14831 (±198.6)	-	2.5 × 10^−6^ (±1.4 × 10^−7^)	0.78 (±6.2 × 10^−3^)	1.4 × 10^−7^ (±1.1 × 10^−8^)	1	-	-	14903.6	1.42 × 10^−7^	98.6
SG_160	10,629 (±1297.4)	203,740 (±2421.2)	18,773 (±2065.1)	-	9.6 × 10^−7^ (±2.2 × 10^−7^)	1	8.5 × 10^−9^ (±1.7 × 10^−9^)	0.9	1.1 × 10^−8^ (±1.8 × 10^−9^)	0.89 (±1.2 × 10^−2^)	233142	1.74 × 10^−9^	99.9
SG_160_ (72h)	-	-	1973 (±56.87)	7145 (±849.4)	5.4 × 10^−6^ (±5.8 × 10^−7^)	0.86 (±1.31× 10^−2^)	-	-		-	1546.07	1.29 × 10^−6^	86.6
Pretreated_Mg	-	-	1041 (±24.36)	305.4 (±18.8)	1.4 × 10^−5^ (±8.9 × 10^−7^)	1	-	-	-	-	236.1	1.46 × 10^−5^	-
Asreceived_Mg	-	-	690.1 (±4.7)	296.4 (±8.2)	2.3 × 10^−5^ (±4.7 × 10^−7^)	1	-	-	-	-	207.3	2.34 × 10^−5^	-

**Table 2:** Normalized bands intensity of the sol–gel without MI (SG) and with MI (SGMI) observed in the region 850–1350 cm^−1^ of the FTIR spectra.

	Normalized Integrated Area					LO/TO	Epoxy/-CH_3_
Peak/cm^−1^	910	1010	1100	1190	1270	-	-
SG	2.54	18.3	16.13	2.69	1	0.88	2.54
SGMI	1.17	21.03	12.68	2.39	1	0.60	1.17
